# Genome Analysis and Phylogenetic Relatedness of *Gallibacterium anatis* Strains from Poultry

**DOI:** 10.1371/journal.pone.0054844

**Published:** 2013-01-24

**Authors:** Timothy J. Johnson, Jessica L. Danzeisen, Darrell Trampel, Lisa K. Nolan, Torsten Seemann, Ragnhild J. Bager, Anders M. Bojesen

**Affiliations:** 1 Department of Veterinary and Biomedical Sciences, University of Minnesota, Saint Paul, Minnesota, United States of America; 2 Department of Veterinary Diagnostic and Production Animal Medicine, College of Veterinary Medicine, Iowa State University, Ames, Iowa, United States of America; 3 Department of Veterinary Microbiology and Preventive Medicine, College of Veterinary Medicine, Iowa State University, Ames, Iowa, United States of America; 4 Victorian Bioinformatics Consortium, Monash University, Clayton, Victoria, Australia; 5 Department of Veterinary Disease Biology, Faculty of Life Science, University of Copenhagen, Frederiksberg C, Denmark; Baylor College of Medicine, United States of America

## Abstract

Peritonitis is the major disease problem of laying hens in commercial table egg and parent stock operations. Despite its importance, the etiology and pathogenesis of this disease have not been completely clarified. Although avian pathogenic *Escherichia coli* (APEC) isolates have been incriminated as the causative agent of laying hen peritonitis, *Gallibacterium anatis* are frequently isolated from peritonitis lesions. Despite recent studies suggesting a role for *G. anatis* in the pathogenesis of peritonitis, little is known about the organism’s virulence mechanisms, genomic composition and population dynamics. Here, we compared the genome sequences of three *G. anatis* isolates in an effort to understand its virulence mechanisms and identify novel antigenic traits. A multilocus sequence typing method was also established for *G. anatis* and used to characterize the genotypic relatedness of 71 isolates from commercial laying hens in Iowa and 18 international reference isolates. Genomic comparisons suggest that *G. anatis* is a highly diverse bacterial species, with some strains possessing previously described and potential virulence factors, but with a core genome containing several antigenic candidates. Multilocus sequence typing effectively distinguished 82 sequence types and several clonal complexes of *G. anatis*, and some clones seemed to predominate among *G. anatis* populations from commercial layers in Iowa. Biofilm formation and resistance to antimicrobial agents was also observed in several clades. Overall, the genomic diversity of *G. anatis* suggests that multiple lineages exist with differing pathogenic potential towards birds.

## Introduction

Avian pathogenic *Escherichia coli* (APEC) has typically been incriminated as the causative agent of laying hen peritonitis [1]. However, evidence suggests that *Gallibacterium anatis* may also play a role in the pathogenesis of this disease [2]. *G. anatis* is a Gram-negative, non-motile, encapsulated, usually β-hemolytic coccobacillus of the *Pasteurellaceae* family that forms grayish, round, semi-transparent colonies. The genus was defined by Christensen et al. [3] and now includes four named species and three additional unnamed genomospecies [4]. Of these, only *G. anatis* is regularly isolated from poultry [5]. This organism is now considered to be an important bacterial agent responsible for decreased egg production in commercial layers, since it infects the reproductive tract and causes pathological changes [2], [6]–[8].

Although great effort has been devoted to understanding the pathogenesis of APEC, the virulence mechanisms possessed by *G. anatis*, and its role as a pathogen, have not been fully elucidated. The prototypic virulent *G. anatis* strain 12656-12 was recently sequenced. Analysis of this sequence identified a RTX-like toxin, GtxA, which contributes to *G. anatis*’ virulence for chickens, and has cytotoxic activity [9]. Furthermore, the secretion system essential for export of GtxA was identified, and the *gtxA* gene was found to be disrupted in non-hemolytic strains of *G. anatis* [10]. Other virulence associated traits among *G. anatis* strains have been identified, such as protease production and hemagglutination, but the underlying genetic traits responsible for these phenotypes have not yet been determined. It has also been proposed that *G. anatis* isolates vary in their virulence potential [11], [12], and amplified fragment length polymorphism (AFLP) has revealed that there is substantial genetic diversity among the *Gallibacterium* isolates dominating among and between successive flocks [13]. The purpose of this study was to generate and compare the genome sequences of virulent and avirulent *G. anatis* isolates to better understand their genetic composition, and to develop a multilocus sequence typing (MLST) procedure for assessing the genetic relatedness of *G. anatis* isolates and their genomic content [14].

## Materials and Methods

### Bacterial Strains and Growth Conditions

All animal experiments were performed in accordance with the Insitutional Animal Care and Use Committee at Iowa State University. Live animal were humanely euthanized using carbon dioxide gas in sealed containers. The strains sequenced in this study included *G. anatis* strains UMN179, 12656-12, and F149^T^. Strain 12656-12 is a well characterized pathogenic strain isolated from the liver of a septicemic chicken in 1981 [7]. Strain F149^T^ is the type strain for *G. anatis* and was isolated from a healthy duck in Denmark in 1979 [3]. Strain UMN179 was isolated in 2007 from a commercial laying hen with peritonitis in Iowa, USA [14]. Additional isolates for MLST analysis were obtained from two commercial egg laying companies in Iowa, USA in 2006 and 2007 involving laying hens from four farm systems and eleven different farms (Table 1). Healthy or diseased birds were received at the Iowa State University Veterinary Diagnostic Laboratory where they were euthanized and necropsied. Swab samples were taken from the following locations: crop, gizzard, small intestine, ceca, cloaca, trachea, lung, liver, spleen, oviduct, and peritoneum. Samples were inoculated onto Remel 5% sheep blood agar and incubated aerobically at 35°C for 24–48 hours. β-hemolytic colonies were verified as Gram-negative via Gram staining, then confirmed as follows via biochemical testing: indole(−), urease(−), trehalose(+), maltose(−), xylose(+/−), arabinose(−), mannitol(+), and sorbitol(+/−) Isolates were further confirmed as *G. anatis* using a PCR-based approach specific for the 16S and 23S rRNA genes, as previously described [15]. After confirmation of the isolates as *G. anatis*, they were stored in BHI broth with 20% w::v glycerol at −80°C until further use. Eighteen previously characterized international *Gallibacterium* reference strains were also used for comparative purposes in MLST analysis, representing the defined biovars and genomospecies of *Gallibacterium* [3], [5], [7].

**Table 1:** *Gallibacterium* strains used for multilocus sequence analysis.

Isolate names	Year of isolation	Flock designation	Flock age at sampling	Flock status at sampling	Number of birds sampled from flock
GA89-GA98	2007	I	37 weeks	Healthy	5
GA99	2007	J	NA	Peritonitis	1
GA104	2007	H	NA	Peritonitis	1
GA105-GA130	2007	A	109 weeks	Healthy	5
GA131-GA141	2007	B	34 weeks	Healthy	5
GA142-GA156	2007	E	15 weeks	Healthy	5
GA155	2006	F	27 weeks	Peritonitis	1
GA157-GA177	2007	D	76 weeks	Healthy	10
GA178	2007	G	NA	Peritonitis	1
UMN179	2007	G	36 weeks	Peritonitis	1
GA180-GA189	2007	C	5 weeks	Healthy	5

### Genome Sequencing and Annotation

Sequencing of strains UMN179, F149^T^, and 12656-12 was performed using 454 Life Sciences pyrosequencing at the University of Minnesota Biomedical Genomics Facility, USA, and at 454 Life Sciences, Branford, CT, USA, respectively. The sequencing of strain UMN179 has been previously described (15). For 12656-12 and F149^T^, one shotgun library each was used and similarly assembled with finishing. Sequences of these genomes are deposited in Genbank under the following accession numbers: UMN179 (CP002667 and CP002668), F149^T^ (PRJNA167529), and 12656-12 (PRJNA167530).

### Comparative Genomics

Annotation of the three *G. anatis* genomes was performed using publicly available tools. Putative coding regions were predicted using GeneMarkS [16]. Gene function was assigned using HMMER3 against Pfam-A 24.0, RPS-BLASTp against CDD, and BLASTp against all microbial proteins [17], [18]. tRNA genes were identified using tRNAscan-SE [19]. rRNA genes were identified using RNAmmer [20]. For analysis of the shared and unique proteins in *G. anatis* genomes sequenced, BlastP was used with a similarity cutoff of 90% identity over 90% of the protein. For genomic regions of difference, whole genome alignments were performed using MAUVE to identify regions present in strain UMN179 but absent from strains F149^T^ and/or 12656-12 [21]. Genomic islands for strain UMN179 were also predicted computationally using IslandViewer [22]. All predicted proteins of UMN179 were analyzed for their predicted subcellular location using PSORT 3.0 [23]. Linear and circular genomic maps were generated using XPlasMap and Circos, respectively [24].

### MLST Analysis

Using the completed sequence of *G. anatis* strain UMN179 and the draft sequences of strains 12656-12 and F149^T^, primers were designed to amplify eight housekeeping genes dispersed throughout the *Gallibacterium* genome (Table 2). PCR was performed using an initial denaturing step at 94°C for 5 min, followed by 25 cycles of 94°C for 30 sec, 55°C for 45 sec, and 72°C for 1 min. The amplified products were electrophoresed in 2% agarose gels at 200 V for 1 hr, stained with ethidium bromide, and visualized under ultraviolet light. Products were bidirectionally sequenced using standard Sanger sequencing reactions. Resulting data were quality assessed and then aligned and trimmed to equal sizes. Concatenated sequences were generated and aligned using ClustalW [25]. Evolutionary distances were computed using the Maximum Composite Likelihood method. A total of 4,172 nucleotide positions were used in the final dataset. Phylogenetic analyses were conducted in MEGA version 4 [26]. Bootstrapping was performed with 500 replicates. The sequences were deposited in Genbank under the following accession numbers: *atpD*, JQ647935–JQ648023; *fumC*, JQ648024–JQ648112; *gyrB*, JQ648113–JQ648201; *mdh*, JQ648202–JQ648290; *recN*, JQ648291–JQ648379; *infB*, JQ648380–JQ648468; *thdF*, JQ648469–JQ648557; and *adk*, JQ648558–JQ648646. An MLST database for the analysis of *Gallibacterium*, based upon this scheme, is available for public use at http://pubmlst.org/gallibacterium/.

**Table 2:** Primer sequences and characteristics of regions used for multilocus sequence analysis.

Gene	Forward primer	Reverse primer	Trimmed length	Variable sites	Informative sites	Percent informative sites
*adk*	CACGGATAATTAAATCTTCG	GCAGGCAAAGGCACACAAGC	439	76	61	13.9
*fumC*	GAATGTGAACGAAGTGGTTG	ACAATCGCATCGTGAGTTGC	512	57	50	9.8
*gyrB*	TGTGCGTTTCTGGCCAAGTC	CGCTCACCAACTGCAGATTC	561	74	61	10.9
*mdh*	ATCCGACTCCTGCGCTTGAAG	ATGCGCGCACAAGTGATAATG	536	57	50	9.3
*recN*	CGGTGCCGGCAAGTCAAT	TTCGCAGTTTCATTTTCCGACAAC	564	70	56	9.9
*thdF*	AATGCCGGTAAATCAAGCCTATTG	GTCCCGTGATCTCGCTGAGTG	595	75	52	8.7
*atpD*	GGTGGTGCGGGTGTAGGTAAAA	GTTGCCGGAGATGGGTCAGTC	495	29	24	4.8
*infB*	TGTAGCAGCGGATGATGGTGTAAT	CGCCGGAAAGCCCTAAAATCTCT	484	64	57	11.8

### Disk Diffusion

Isolates were tested for susceptibility to seven antimicrobial agents by an agar disk diffusion method (Kirby-Bauer) according to the CLSI MIC Interpretative Standards using Mueller–Hinton agar plates [27]. Disks containing the following antimicrobial agents were used: ampicillin (AM, 10 µg), gentamicin (GM, 10 µg), nalidixic acid (NA, 30 µg), streptomycin (S, 10 µg), tetracycline (TE, 30 µg), sulfisoxazole (G,.25 µg) and trimethoprim (TMP, 1.2 µg) (BD, Franklin Lakes, NJ). The following *E. coli* strains were used as positive controls: ATCC 25922 and APEC O1.

### Biofilm Assay

Biofilm formation was quantified in 96-well polystyrene microtiter plates (Falcon Microtest 353072; Becton Dickinson, Franklin Lakes, NJ, USA), based on previously described methods [28], [29]. Overnight BHI broth cultures of the strain to be tested were diluted 1∶100 in BHI (Difco). A volume of 200 µl aliquots of each dilution was then dispensed into a microtiter plate well. Negative control wells contained uninoculated medium, and each strain was tested in triplicate. Plates were inoculated aerobically without shaking at 37°C for 24 h. Contents of the plates were then decanted, and plates were washed with sterile double distilled water. Microplates were then stained with 200 µl of 0.1% Crystal Violet for 30 min, washed four times with ddH2O to remove excess stain, and air dried for 1 h. After drying, adherent cells were resolubilized with 200 µl of an 80∶20 solution of ethanol and acetone. A volume of 150 µl of this solution was then transferred to a new microtiter plate, and the OD_600_ was measured using an automated ELx808 Ultra MicroPlate Reader (BioTek Instruments, Winooski, VT, USA). All tests were carried out in triplicate, and the results were averaged. Based on the OD produced by bacterial biofilms, strains were classified into the following categories: non, weak, moderate or strong biofilm producer, based on a previously described method [29], [30]. Briefly, the cutoff OD (ODc) was defined as three standard deviations above the mean OD of the negative control. Strains were classified as follows: OD<ODc = no biofilm production; ODc<OD<(2 · ODc) = weak biofilm producer (2 · ODc)<OD<(4 · ODc) = moderate biofilm producer; and (4 · ODc)<OD = strong biofilm producer.

## Results and Discussion

### Overview of the *G. anatis* Genomes

The completed sequence of strain UMN179 was previously generated and used here for genomic comparisons (15). A total of 390,760 reads were used to draft assemble F149, resulting in 128 contigs yielding a draft assembled size of 2,403,410 bp at an average of 31-fold genome coverage. A total of 606,921 reads were used to draft assemble 12656-12, resulting in 115 contigs yielding a draft assembled size of 2,540,739 bp at an average of 26-fold genome coverage.

**Figure 1:**
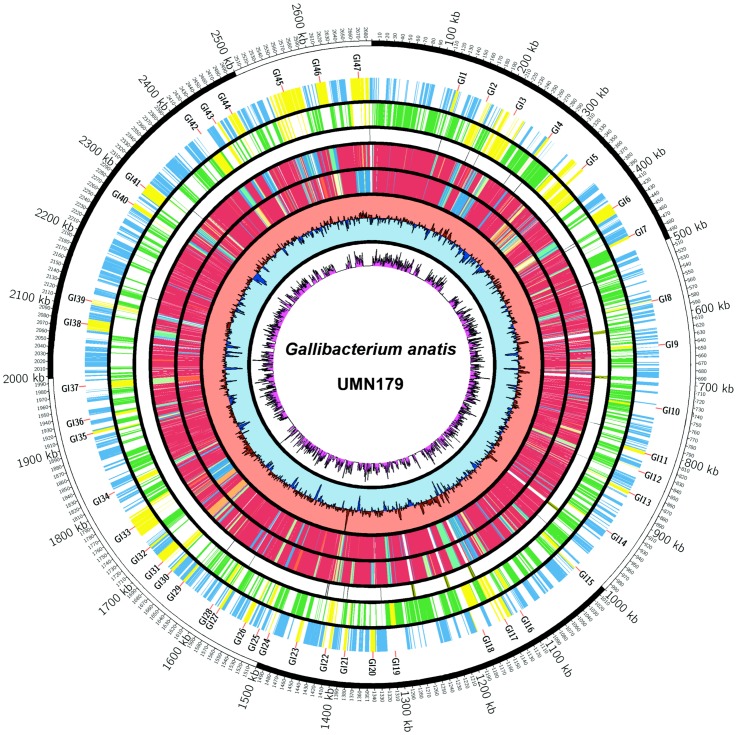
Circular representation of the *Gallibacterium anatis* UMN179 chromosome. The outer circle depicts scale in kilobase pairs. The next circle designates genomic regions present in UMN179 but absent from F149^T^. Circles 3–4 depict coding regions (blue = positive strand; green = minus strand; yellow = island genes). Circle 5 depicts tRNA and rRNA loci. Circles 6–7 depict the presence of coding regions in UMN179 compared to 12656-12 and F149, respectively, based upon amino acid similarity heatmap ranging from red (high similarity) to blue (no similarity). Circle 8 depicts G+C content relative to the genome average of 40%, with salmon spikes above 40% and light blue spikes below 40%. Circle 9 depicts single nucleotide polymorphism abundance within the core genes.

**Figure 2:**
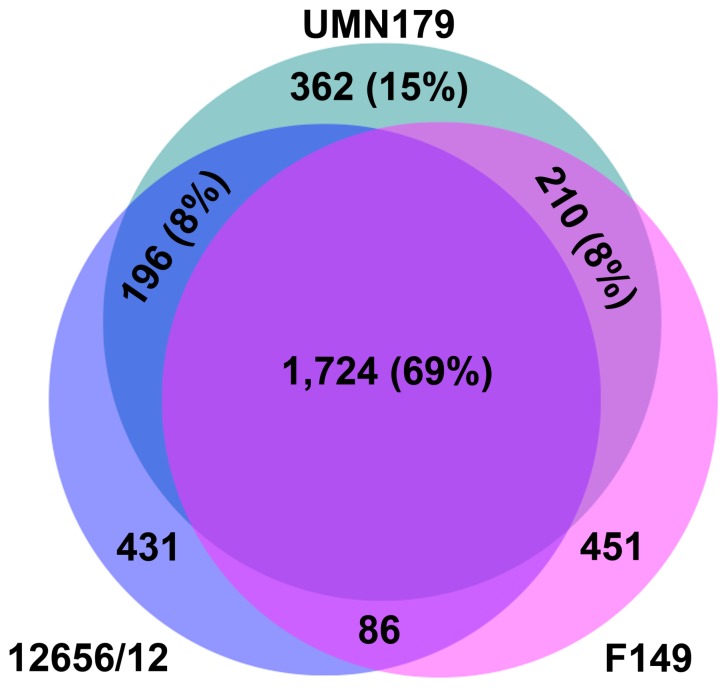
Venn diagram depicting shared and unique proteins among *Gallibacterium anatis* strains UMN179, F149^T^, and 12656-12. Percentages are relative to UMN179 total predicted proteins.

The complete sequence of UMN179 was compared to the draft sequence of F149^T^ in an effort to identify genomic regions present in strain UMN179, greater then 2,000 bp, that were not present in F149^T^, excluding insertion sequence element insertions not involving additional genetic material (Fig. 1 and Table 3). In total, 47 genomic regions of difference were identified, 18 of which were also present in 12656-12. These regions ranged from 2.1 to 55.6 kb in size. Four of the regions contained prophage-like elements. Analysis of the predicted proteins of the three sequenced strains using a 95% amino acid similarity cutoff revealed that 69% of the predicted proteins of the three sequenced genomes were shared, 15% of the proteins of UMN179 were unique compared to F149^T^ and 12656-12, and 8% of the proteins were shared with UMN179 and F149^T^ or 12656-12, respectively (Fig. 2). This is roughly similar to what has been previously observed for *E. coli* [31], suggesting that the proportional pangenome size of *G. anatis* might be similar to that of *E. coli* and other Gammaproteobacteria.

**Table 3:** Genomic regions of difference present in *Gallibacterium anatis* UMN179 but absent from F149^T^. Name designations refer to that used in Fig. 1.

Name	Start	Stop	Size	Presence in 12656/12	Key Features	G+C content
GI1	127673	131511	3838	present	HTH-type transcriptional regulator	35.6
GI2	167769	183066	15297	absent	Major facilitator transporter; LysR transcriptional regulator	37.7
GI3	201381	240582	39201	absent	Prophage	42.2
GI4	280789	285316	4527	absent	Bacteriocin synthesis and ATPase proteins	32.5
GI5	322796	378439	55643	similar	Integrative conjugative element	37.5
GI6	416901	434462	17561	absent	Hemagglutinin and two-partner secretion protein	41.1
GI7	472215	476572	4357	present	Putative ABC iron transport system	38.2
GI8	574481	578199	3718	absent	Lipoprotein and surface protein with XRE family transcriptional regulator	35.4
GI9	640054	644602	4548	present	Major facilitator transporter, oxidase, and decarboxylase	27.4
GI10	735125	738340	3215	present	Unknown function	30.2
GI11	803433	808950	5517	present	Chaperone-usher fimbrial system	35.2
GI12	829667	838190	8523	absent	Putative hemagglutinin	39.8
GI13	865478	872469	6991	similar	Chaperone-usher fimbrial system	37.3
GI14	933531	935711	2180	absent	Putative curli production operon	33.5
GI15	1002230	1009409	7179	present	Sulfur-related oxygen sensing system	33.3
GI16	1108629	1114870	6241	absent	Putative hemagglutinin	39
GI17	1126848	1148930	22082	absent	Unknown function	38.7
GI18	1164994	1183251	18257	absent	Group II intron and large repeat protein	43.7
GI19	1304418	1312738	8320	absent	Type I restriction modification system	35.7
GI20	1338022	1345999	7977	present	Fucose-like sugar utilization system	38.5
GI21	1380749	1386769	6020	present	Sugar phosphate transport and sensor-kinase system	29.9
GI22	1403996	1420490	16494	absent	Two putative metal resistance systems	44.2
GI23	1453434	1459320	5886	absent	Putative hemagglutinin	38.7
GI24	1496265	1502879	6614	absent	Putative autotransporter	37.3
GI25	1513492	1518412	4920	absent	Unknown function	24.8
GI26	1533192	1542954	9762	absent	Putative hemagglutinin	40.4
GI27	1579940	1584951	5011	absent	Major facilitator transporter, phospholipase	37.2
GI28	1591409	1596421	5012	present	Unknown function	29.2
GI29	1647413	1651284	3871	present	Ribose-like transport system	40
GI30	1671073	1676071	4998	present	Glucitol/sorbitol-like phosphotransferase system	35.3
GI31	1681412	1703052	21640	absent	Type II restriction modification system, toxin/antitoxin, hemagglutinin	38.8
GI32	1719472	1722677	3205	present	Unknown function	37.6
GI33	1741002	1776977	35975	absent	Prophage	44.1
GI34	1814490	1822810	8320	present	Myo-inositol catabolism	39
GI35	1908758	1915189	6431	present	Tripartite ATP-independent periplasmic transporter system	35
GI36	1931387	1936078	4691	present	Tripartite ATP-independent periplasmic transporter system	40.3
GI37	1977419	1989255	11836	absent	Putative hemagglutinin	40.6
GI38	2067730	2084075	16345	shared	Putative hemolysin/toxin and secretion system	34.7
GI39	2106314	2115266	8952	absent	IS10-Tet with novel region	40.1
GI40	2264065	2271933	7868	present	Putative ABC transport system	41.1
GI41	2290662	2306895	16233	absent	Capsular biosynthesis proteins	30.2
GI42	2406946	2422424	15478	absent	Hemagglutinin and two-partner secretion protein	37.9
GI43	2437460	2446646	9186	present	Heme-binding system *hxuABC*;heme-iron utilization system *hutXZ*	38.9
GI44	2462454	2477902	15448	partial	CRISPR locus	35.6
GI45	2528572	2578722	50150	absent	Prophage	39.8
GI46	2602084	2617739	15655	absent	Hemagglutinin	37.4
GI47	2651193	2683490	32297	absent	Prophage	42.8

Strain UMN179 contained a 6,804-bp plasmid, pUMN179. The replication gene of this plasmid exhibited closest similarity to small plasmids from *Pasteurella multocida* strain BB1034 and *Mannheimia haemolytica* strain OVINE. The plasmid also contained genes encoding a TraG-like protein and a prophage-like integrase. No antibiotic resistance genes were identified on pUMN179 and no plasmid-like contigs were identified in strains 12656-12 or F149.

The G+C content of UMN179 was approximately 40%. The G+C content in UMN179 was analyzed using a 1,000 bp sliding scale, which demonstrated that numerous genomic islands contained a higher or lower G+C content than the average, further suggesting horizontal transfer of these regions (Fig. 1). Single nucleotide polymorphisms were also examined between UMN179, F149^T^, and 12656-12 within the core genome (Fig. 1). Regions of difference not present in all three sequenced strains were excluded from this analysis. SNPs were mostly evenly distributed throughout the core genome and coding regions with a higher density of SNPs did not exhibit any discernable patterns, such as similarity in function or subcellular location.

**Figure 3:**
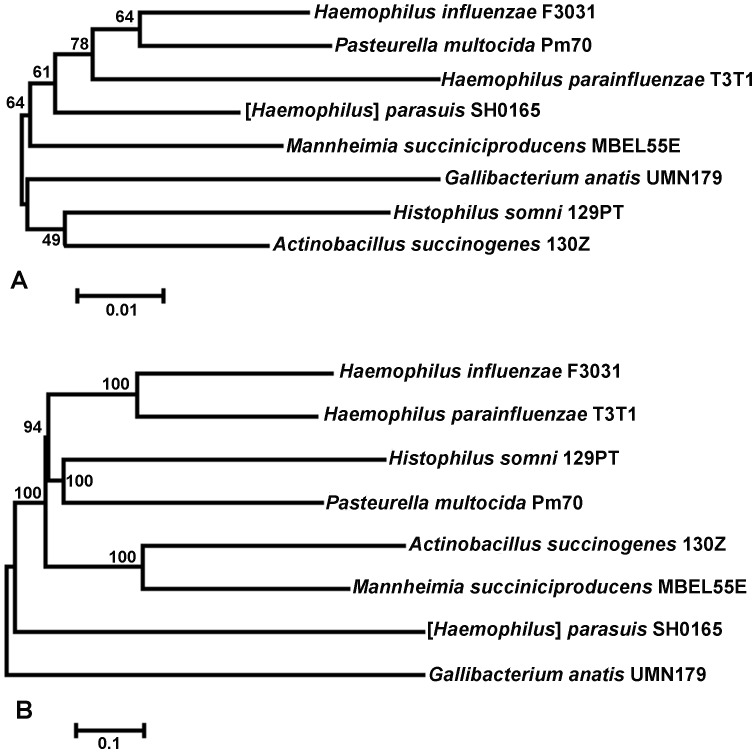
Inferred phylogeny of eight completed bacterial genomes. Panel A: Inferred phylogeny of eight completed bacterial genomes using their 16S rRNA sequences. Data was analyzed using the Maximum Likelihood method based on the JTT matrix-based model conducted in MEGA5. There were a total of 1,515 positions in the final dataset and 100 bootstrap replicates were included. Panel B: Inferred phylogeny of eight completed bacterial genomes using whole genome alignment. Alignments were conducted in MAUVE [21] and SNPs within conserved regions were extracted. Data was analyzed using the Maximum Likelihood method based on the JTT matrix-based model conducted in MEGA5. There were a total of 98,659 positions in the final dataset and 100 bootstrap replicates were included.

Whole genome alignments were performed between the completed sequence of strain UMN179 and seven other completed bacterial genomes. Inferred phylogeny was then constructed based upon SNPs within conserved regions of the genomes, and compared to inferred phylogeny using their 16S rRNA genes (Fig. 3). In both comparisons, *G. anatis* strain UMN179 was the most divergent genome compared to its closest available completed sequences in the database. However, this was accentuated in the whole genome alignments compared to 16S rRNA comparisons, and whole genome alignments painted a slightly different picture on the relatedness of these genomes. Specifically, whole genome alignment suggests early divergence of *Gallibacterium* with *Haemophilus parasuis*, while based on 16S rRNA analysis *Gallibacterium* clusters with *Histophilus somni* and *Actinobacillus succinogenes*.

### Putative Virulence Factors

Few virulence factors of *G. anatis* have been described. One key virulence factor of *G. anatis* is its RTX-like toxin named GtxA [9], and its associated secretion system [32]. All three genomes sequenced were found to contain *gtxA* in similar genomic locations (UMN179_1781). However, as previously described, in strain F149^T^ this protein is disrupted by an insertion sequence, likely explaining its lack of hemolytic activity. However, it has been shown that isolates lacking hemolytic ability are not phylogenetically related [32]. Thus, it appears that such insertional inactivations of GtxA have occurred on separate occasions resulting in convergent evolution of non-hemolytic *G. anatis*. The secretion system for GtxA is *gtxEBD* [32]. This system is also found within the core genome of *G. anatis* at similar locations in all sequenced strains (UMN179_1226–1228).

Previous work has identified secreted metalloproteases in *G. anatis* with proteolytic activity capable of degrading chicken IgG [33]. We identified one predicted protein (UMN179_900) that contained a metal-dependent endonuclease domain, was predicted to be extracellular, and was present in all sequenced *G. anatis*. Additionally UMN179_1874 was identified as a zinc metalloprotease that was present in all sequenced genomes, and UMN179_572 was identified as an ATP-dependent metalloprotease present in all sequenced genomes. These proteins could be responsible for the proteolytic capability of *G. anatis*.

### Adhesins

The genome of strain UMN179 was found to contain a number of predicted adhesins. Three products, UMN179_416, UMN179_2244, and UMN179_2445, were similar to one another and possessed hemagglutinin activity and pre-toxin domains. Each of these also appears to be a filamentous hemagglutinin-like protein linked to a two-partner secretion system, with adjacent proteins containing domains related to secretion and activation of hemolysins/hemagglutinins. These three predicted proteins were unique to UMN179, as compared to 12656-12 and F149^T^. Strain UMN179 also contained six predicted hemagglutinin/hemolysin/calcium-binding-like proteins of varying lengths and similarities with one another. All were unique and shared low similarity with the other predicted proteins in UMN179, but all were more similar to one another than to other predicted proteins in the NCBI database. Interestingly, many of these genes were adjacent to truncated versions of predicted hemagglutinins. In strains 12656-12 and F149^T^, similar predicted proteins were identified in some cases. For example, UMN179_1030 and UMN179_1346 shared 45% and 80% identity, respectively, with predicted proteins from 12656-12 in the same genomic locations. UMN179 also possessed several additional predicted proteins with YadA domains, one putative autotransporter/adhesin (UMN179_1565), and one putative serine protease (UMN179_1925).

### Fimbriae

**Figure 4:**
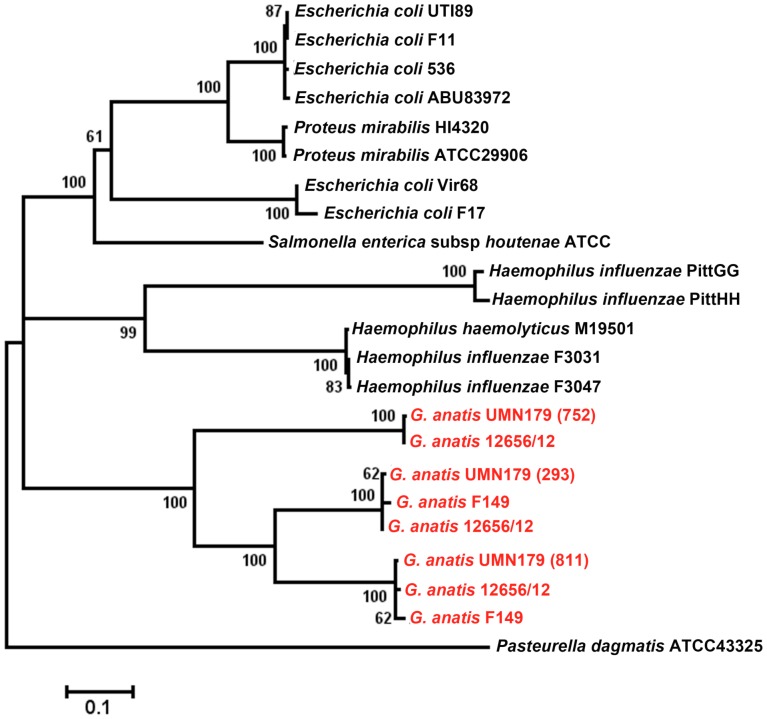
Inferred phylogeny of the fimbrial usher proteins of UMN179. Data was analyzed using the Maximum Likelihood method based on the JTT matrix-based model conducted in MEGA5. There were a total of 666 positions in the final dataset and 500 bootstrap replicates were included.

Strain UMN179 contained three predicted chaperone/usher fimbrial loci. All were four-gene F17-like systems encoding apparently intact chaperone and usher proteins. Two fimbrial systems (UMN179_293–295 and UMN179_809–812) were present in all three sequenced genomes, whereas an intact version of the remaining system (UMN179_750–753) could only be identified in the genomes of UMN179 and 12656-12. A truncated version could, though, be identified in F149^T^. This system lacked the genes encoding the usher and adhesin protein, and moreover, the chaperone-encoding gene was only partly represented. We compared the fimbrial ushers for each of these systems with existing fimbrial systems from the NCBI database (Fig. 4). From amino acid alignments of these proteins, each protein was most similar to those from the same locus (in different isolates) and same species (in different loci), but all predicted proteins from *G. anatis* were most similar to one another. This is suggestive that the F17-like fimbrial systems in *G. anatis* have arisen through duplication of these systems resulting in multiple paralogs following speciation. Interestingly, a transposon was located just upstream the truncated version of the fimbrial locus in F149^T^. A similar transposon was also found in the region upstream the locus-homologs in strain UMN179 and 12656-12, respectively, further suggesting horizontal gene transfer of paralogs within *G. anatis*.

### Capsular Polysaccharide Components

The genomes of UMN179 and F149^T^ did not contain detectable capsular polysaccharide biosynthesis genes. However, 12656-12 contained a putative capsular biosynthesis gene cluster inserted within genes similar to UMN179_44 and UMN179_45. This gene cluster contained 13 coding regions designated *lipA2*-*lipA1*, *gcbG*-*A*, and *gexD*-*A*. Capsular components of *G. anatis* have not yet been described, but some *G. anatis* do possess a capsular structure (A.M. Bojesen, unpublished results).

### CRISPRs

Clustered regularly interspaced short palindromic repeats (CRISPRs) are arrays or repeats common among bacteria and archaeae that provide protection against foreign DNA [34]. These loci are identified by their possession of *cas* gene clusters, and the presence of non-contiguous direct repeats separated by variable spacer regions. Several CRISPR loci were identified within the three *G. anatis* genomes sequenced [35]. The first CRISPR locus was found between UMN179_2281 and UMN179_2300 and was present in UMN179 and 12656-12 on GI44 (Table 3). This region contained the genes *csm1*-*csm2*-*csm3*-*csm4*-*csm5*-*cas6*-*csx16*-*csm6*-*csx1*-*cas2*-*cas1*-*cas2*, with 31 direct repeats in UMN179 and 15 direct repeats in 12656-12. A second CRISPR locus was identified between UMN179_2337 and UMN179_2343 and was present in all three genomes. This locus contained *cas3*-*cys4*-*cys3*-*cys2*-*cys1* and had 8 direct repeats in UMN179, and 7 direct repeats each in F149^T^ and 12656-12. A third locus was present in strains F149^T^ and 12656-12, but absent in UMN179, situated between UMN179_173 and UMN179_174. This region contained *cas3*-*cas5*-*csd1*-*csd2*-*cas4*-*cas1*-*cas2* and had 6 direct repeats in 12656-12 and 48 direct repeats in F149^T^. None of the spacers within any of the three strains shared significant nucleotide similarity with spacers of other strains. Similarly, none of the three CRISPR loci genes shared significant amino acid similarity with genes from other loci. The presence of all CRISPR loci in multiple strains of the three sequenced genomes suggest that they were acquired by ancestral strains and have rapidly evolved to acquire different subsets of spacer elements. Since none of the strains sequenced here are very closely phylogenetically related (see below), it is not surprising that they differ in their spacer compositions. It remains to be determined if CRISPR loci in *Gallibacterium* can be used as phylogenetic tools and as a means of predicting the propensity to acquire foreign DNA.

Other loci of importance for DNA uptake were encoded in the competence regulon, which was present in all three strains, as previously described [36]. Interestingly, F149^T^ exhibited a transformation frequency that was three orders of magnitude lower than 12656-12 [36]. Thus, although most *G. anatis* strains studied appear to be naturally competent, this ability seems differentially regulated between strains [36].

### Integrative Conjugative Elements (ICE) and Resistance Elements of *Gallibacterium*

**Figure 5:**
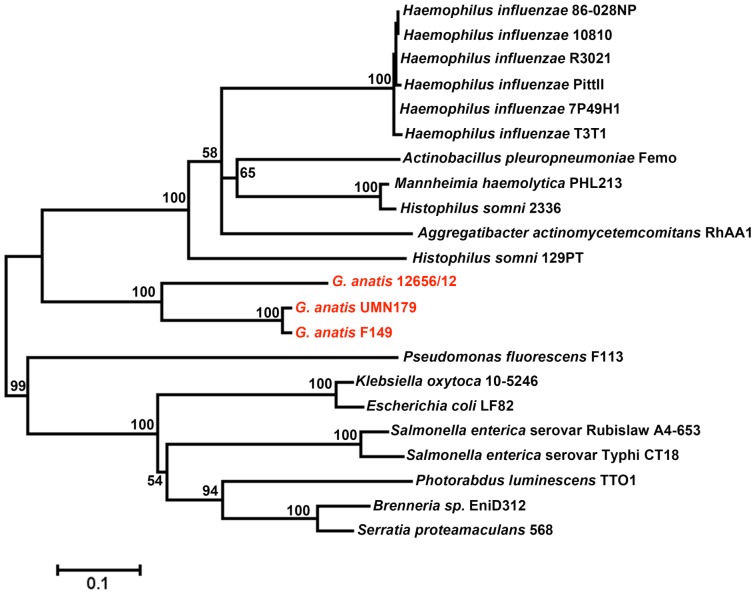
Inferred phylogeny of the TopB proteins of integrative conjugative elements (ICEs) of UMN179, F149, and 12656-12 and similar ICEs in the NCBI database. Data was analyzed using the Maximum Likelihood method based on the JTT matrix-based model conducted in MEGA5. There were a total of 596 positions in the final dataset and 500 bootstrap replicates were included.

Integrative conjugative elements (ICEs) are well-described entities that are able to integrate and excise from bacterial genomes [37]. ICEs have some trademark signatures, including a site-specific integrase, transfer genes, and genes regulating excision and transfer. ICEs also often possess accessory elements conferring antimicrobial resistance. All three sequenced *G. anatis* strains contained single but distinct ICEs within their genomes. These ICE was integrated within the tRNA^Leu^ in strains UMN179 (UMN179_316 through UMN179_382), similar to ICE*Pmu1* found within the genome of *Pasteurella multocida* strain 36950 [38]. In strain 12656-12, its ICE element was integrated into tRNA^Met^. We were unable to discern the genomic location of the ICE of strain F149. These ICEs did not appear to harbor any antimicrobial resistance genes but did contain YadA domain-containing coding regions with similarity to a hemagglutinin/invasin (Fig. S1). The presence of distinct ICEs in multiple locales within the three *G. anatis* genomes suggests that these may play an important and active role in their evolution. Based upon phylogenetic analysis of the *topB* gene of the ICEs in *G. anatis*, it seems that the ICEs of *G. anatis* are more closely related to one another than those of closely related species (Fig. 5).

It was previously reported that the *tet*(31) resistance determinant is common among *G. anatis* [39]. In strain UMN179, this region is absent but another antimicrobial resistance island is present. This island is flanked by IS*10* transposase elements and encodes an AraC family transcriptional regulator annotated in some cases as TetD, a TetR family transcriptional regulator annotated in some cases as TetC, a major facilitator superfamily protein annotated in some cases as TetA (56% amino acid similarity to TetA(31)), a second TetR transcriptional regulator (64% amino acid similarity to TetR(31)), an ArsR family transcriptional regulator, an amino acid binding ACT domain protein, and a truncated sodium/glutamate symporter. Since strain UMN179 is resistant to tetracycline, it is likely that this region plays a role in tetracycline resistance and represents a mechanism in addition to *tet*(31) by which *G. anatis* is able to resist tetracycline.

### Prediction of Antigenic Candidates of *G. anatis*

Predicted subcellular locations of the proteins of UMN179 were assessed using PSORTb version 3.0 in an effort to determine possible antigenic candidates that are surface exposed proteins (Table 4). In total 54 (2.2%) outer membrane proteins and 21 (0.8%) extracellular proteins were predicted. Predicted outer membrane proteins included putative autotransporter/adhesins, fimbrial usher proteins, hemolysin secretion proteins, the secretion system for the RTX-like toxin GtxA, iron regulated proteins, and other predicted outer membrane proteins of unknown function (Tables S1 Tables S1 and S2). Many of these proteins were conserved among the *G. anatis* strains sequenced, suggesting their potential as future antigenic candidates for vaccine production.

**Table 4:** Prediction subcellular localization of *Gallibacterium anatis* UMN179 proteins using PSORTb 3.0.

Predicted localization	Count	Percent
Cytoplasmic	1197	48.0
Cytoplasmic membrane	542	21.7
Extracellular	21	0.8
Outer membrane	54	2.2
Periplasmic	61	2.4
Unknown/multiple localization	619	24.9

### MLST of *G. anatis*

**Figure 6:**
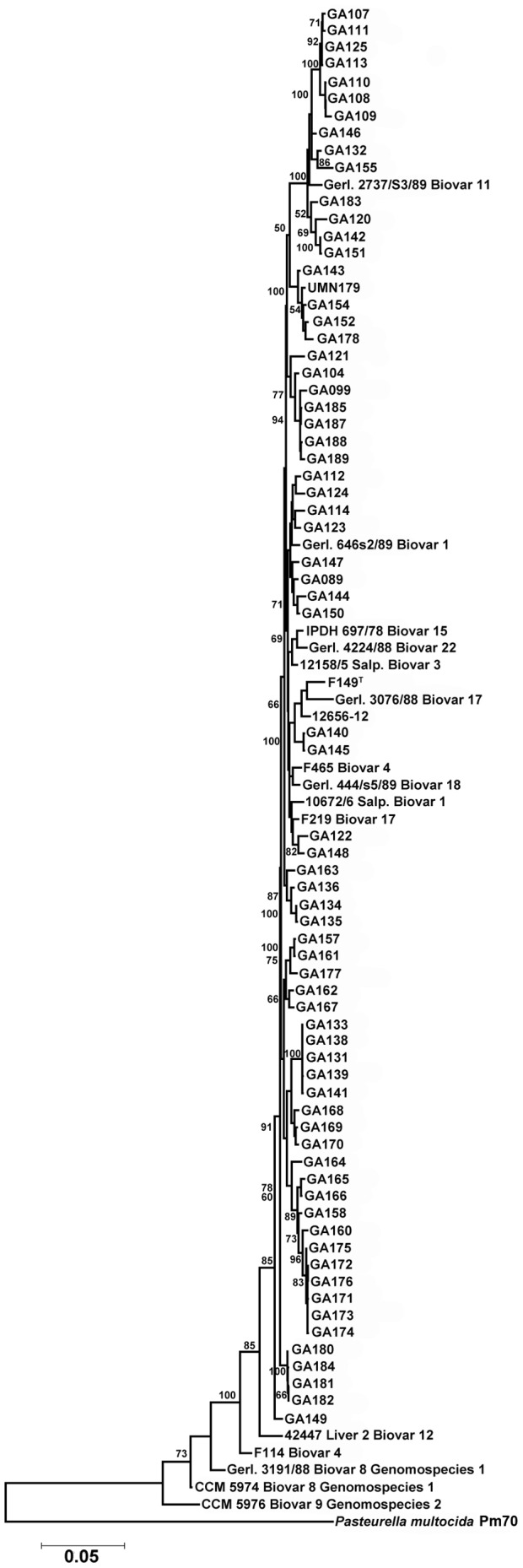
Inferred phylogeny of *Gallibacterium anatis* using concatenated sequences for eight housekeeping genes. Data was analyzed using the Maximum Likelihood method based on the Hasegawa-Kishino-Yano model in MEGA5. A total of 4,172 positions were used in the final dataset and 100 bootstrap replicates were included.

**Figure 7:**
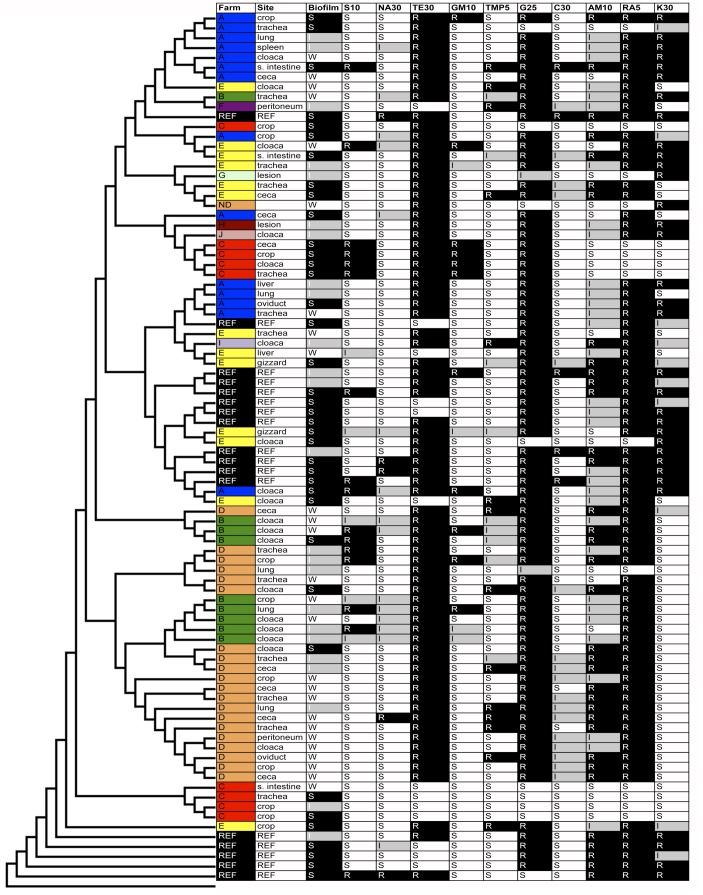
Farm designations, isolation sites, biofilm forming capabilities, and antimicrobial susceptibilities of *Gallibacterium anatis* examined in this study. Farms are colored by source. Biofilm data are classified by strong (S), intermediate (I), or weak (W) biofilm formers. Antimicrobial susceptibilities are depicted as resistant (R), intermediate (I), or susceptible (S) using disk diffusion. Abbreviations for antibiotics used are S10 = streptomycin, NA30 = nalidixic acid, TE30 = tetracycline, GM10 = gentamicin, TMP5 = trimethoprim, G25 = sulfisoxazole, C30 = chloramphenicol, AM10 = ampicillin, RA5 = rifampin, and K30 = kanamycin. The dendrogram was constructed as described in Fig. 6 and is identical order to Fig. 6.

In an effort to better appreciate the phylogenetic diversity of *G. anatis*, 71 isolates belonging to four farm systems and collected at ten timepoints during 2006–2007 were examined for their genetic relatedness using a MLST scheme developed here. Some isolates came from differing body sites within the same bird, while others came from different birds within the same flock (Table 1). Eighteen reference strains belonging to various subgroups of *G. anatis* were also included for comparison purposes, including the isolates sequenced here. Assignment of sequence type (ST) was performed and used to develop an MLST database (http://pubmlst.org/gallibacterium/). In total, 82 STs were identified from the 89 isolates examined, underscoring the genetic diversity of the isolates examined. The dendrogram constructed based upon concatenated gene sequences revealed significant diversity among all of the isolates examined, with the greatest similarities between isolates belonging to the same farm systems and/or timepoints examined (Figs. 6 and 7). The location of isolation within the bird did not correlate with the dendrogram, as isolates from multiple bird sites were scattered throughout the dendrogram. Isolates were grouped by farm source. Group ‘A’ isolates (n = 14) were found mostly in one predominant lineage (n = 7) with the remainder distributed throughout the dendrogram. Group ‘B’ isolates (n = 9) were mostly found within two closely related lineages (n = 8). Similarly, group ‘C’ isolates (n = 9) also clustered into two distinct and unrelated groups. Nearly all group ‘D’ isolates (n = 20) were found in several closely related clusters that were distinct from the rest of the isolates examined. In contrast, group ‘E’ isolates (n = 13) were more diverse and spread throughout the dendrogram. Notably, most reference isolates examined clustered closely, while additional clinical isolates examined expanded the diversity of the dendrogram considerably.

Previously, several approaches have been used to examine the genetic diversity of *Gallibacterium* isolates. Bojesen et al used AFLP to examine fifty *Gallibacterium* isolates belonging to 29 flocks in California, USA [13]. They demonstrated substantial genetic diversity within the *Gallibacterium* genus, differentiation of isolates belonging to *Gallibacterium* genomospecies 1 and 2 from formal *G. anatis* isolates, and the identification of dominant clones that persisted in the same operation throughout a 6-year period. Other typing approaches have been used to characterize *Gallibacterium*, including DNA:DNA hybridization,16S rRNA analysis, and MLST, that also clearly separate *G. anatis* from *Gallibacterium* genomospecies 1 and 2 [3], [4], [40]. However, correlations between phenotypic biovar and phylogeny within *G. anatis* are less clear. We can conclude from the development of the current MLST approach that the previously identified *G. anatis* biovars are representative of much of the genetic diversity within *G. anatis*, but diversity within this genus is even greater than that of currently defined biovars. Also, we conclude that dominant clones of *G. anatis* exist in some farm operations, while in other farms the populations are more diverse. The publicly available MLST database for *Gallibacterium* will provide a worldwide opportunity to further study such strain diversity.

Isolates assessed by MLST were also compared for their ability to form biofilms on a plastic substrate and their susceptibility to ten antimicrobials using disk diffusion (Fig. 7). Biofilm formation was classified as ‘strong,’ ‘intermediate,’ or ‘weak’ based upon a previously used crystal violet assay [30]. Patterns were observed when relating biofilm formation to inferred phylogeny. For example, several clusters that contained previously characterized reference strains were classified as strong or intermediate biofilm formers, as were most isolates belonging to the cluster of isolates containing UMN179. Strains F149^T^, CCM5974 and 12158/5 salp. have previously been shown to be strong at biofilm formation, which to some degree validates the current results [41]. Many isolates from the Iowa collection, though, were weak biofilm formers that belonged outside of the aforementioned clusters. We are unable at this point to determine if certain genomic regions are associated with a stronger ability to form biofilm, however there does appear to be a phylogenetic correlation with strong versus weak biofilm formers.

Most isolates examined were resistant to tetracycline, sulfisoxazole, and rifampin, while some were also resistant to kanamycin, and these isolates tended to cluster together on the dendrogram. The genetic basis for tetracycline resistance has been described above, but mechanisms of resistance to these other antimicrobials are currently unknown. The presence of entire lineages resistant to certain antimicrobials suggests that some of these traits are well established in such lineages and are likely encoded within their core chromosomes rather than within accessory genomic islands or transmissible plasmids. The presence of resistance traits on islands was previously suggested by Bojesen *et al*., [42] who showed that distinct resistance phenotypes could be identified in genotypically different isolates from unrelated flocks. Therefore, horizontal gene transfer likely plays an additional role in the acquisition of resistance to antimicrobial agents by *Gallibacterium*.

In conclusion, this study defined the initial core and accessory genomes of *G. anatis* and identified a number of loci with the potential to play a role in the pathogenesis of this bacterium. Substantial genomic differences among the strains examined suggest that *G. anatis*, a commensal organism in avian species, comprise a diverse group of isolates that differ in their pathogenic potential. Future work is necessary to fully understand the role of the genomic repertoire of *G. anatis* relative to its disease-causing ability.

## Supporting Information

Figure S1 Linear map of the integrative conjugative element of *Gallibacterium anatis* strain UMN179.
**(TIF)**

Table S1 Predicted proteins localized to the outer membrane of *Gallibacterium anatis* strain UMN179.
**(PDF)**

Table S1 Predicted proteins localized as extracellular in *Gallibacterium anatis* UMN179.
**(PDF)**
