# Supplementary figures and images for: Genome Analysis and Phylogenetic Relatedness of Gallibacterium anatis Strains from Poultry
Source: PLoS One. 2013 Jan 24;8(1):e54844. doi: 10.1371/journal.pone.0054844 (PMC3554606; doi:10.1371/journal.pone.0054844)

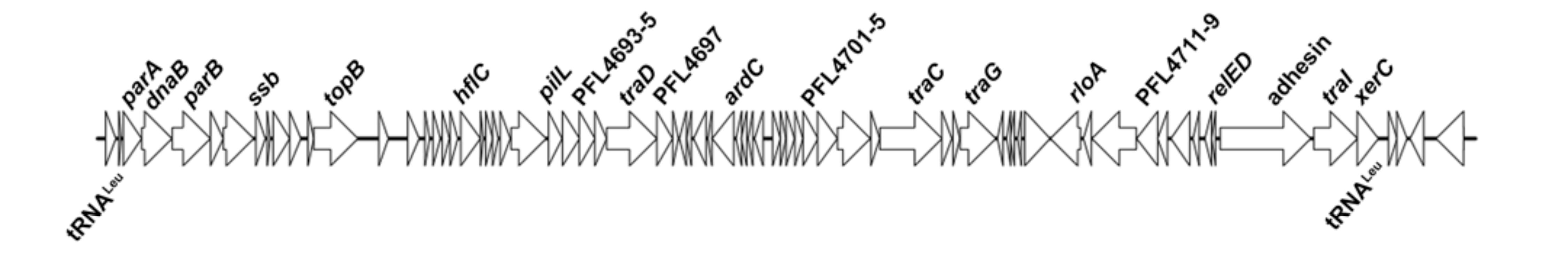

Supplement: Figure S1 — Linear map of the integrative conjugative element of Gallibacterium anatis strain UMN179. (TIF) [file pone.0054844.s001.tif]
