# Supplementary material for: Genome Analysis and Phylogenetic Relatedness of Gallibacterium anatis Strains from Poultry
Source: PLoS One. 2013 Jan 24;8(1):e54844. doi: 10.1371/journal.pone.0054844 (PMC3554606; doi:10.1371/journal.pone.0054844)
Supplement: Table S1 — Predicted proteins localized to the outer membrane of Gallibacterium anatis strain UMN179. (PDF) [file pone.0054844.s002.pdf]

Table S2. Predicted proteins localized as extracellular in *Gallibacterium anatis* UMN179.

**UMN179 Gene Locus**	**Conservation in sequenced *G. anatis***	**Protein Name**
213	UMN179 and F149	Phage tail fiber repeat protein
295	All	Putative fimbrial protein
514	All	Patatin-like phospholipase
750	UMN179 and 12626/12	Putative fimbrial protein
776	UMN179	Hypothetical protein
809	All	Putative fimbrial protein
900	All	Putative DNA-binding/iron metalloprotein/AP endonuclease
1081	UMN179	Hemolysin-type calcium-binding repeat protein
1378	UMN179	Putative autotransporter
1413	UMN179	Putative toxin/calcium binding protein
1508	All	Opacity-associated protein
1565	UMN179	Putative hemagglutinin
1572	All	Glycerophosphodiester phosphodiesterase
1638	UMN179	Phage tail fiber repeat protein
1717	All	Nucleoside diphosphate kinase
1728	All	electron transport complex protein RnfC
1781	All	RTX toxin GtxA
1819	All	Putative major pilin subunit
1925	UMN179	Putative toxin/calcium binding protein
2208	All	Hypothetical protein
2261	UMN179	Putative hemagglutinin
