# Supplementary material for: Genome Analysis and Phylogenetic Relatedness of Gallibacterium anatis Strains from Poultry
Source: PLoS One. 2013 Jan 24;8(1):e54844. doi: 10.1371/journal.pone.0054844 (PMC3554606; doi:10.1371/journal.pone.0054844)
Supplement: Table S1 — Predicted proteins localized as extracellular in Gallibacterium anatis UMN179. (PDF) [file pone.0054844.s003.pdf]

Table S1. Predicted proteins localized to the outer membrane of *Gallibacterium anatis* strain UMN179.

**UMN179 Gene Locus**	**Conservation in sequenced *G. anatis***	**Protein Name**
2	UMN179 and 12656/12	Putative autotransporter/adhesin
70	All	Outer membrane protein OmpA
88	All	Murein transglycosylase A
96	All	Putative outer membrane protein
143	All	Copper/silver efflux system outer membrane protein CusC
250	All	Paraquat-inducible protein B
293	All	Putative fimbrial usher protein
307	All	Outer membrane protein
317	All	Hypothetical protein
415	UMN179	Hemolysin secretion/activation protein
416	UMN179	Putative hemagglutinin
418	UMN179	Hypothetical protein
420	UMN179	Hypothetical protein
438	All	Conserved hypothetical protein
496	UMN179	Hypothetical protein
506	All	Conserved hypothetical protein
549	UMN179	Opacity family porin
576	All	Hypothetical protein
589	UMN179	Putative adhesin
631	All	Hypothetical protein
705	UMN179	Putative adhesin
752	All	Putative fimbrial usher protein
811	All	Putative fimbrial usher protein
870	All	VacJ-like lipoprotein
948	All	Opacity family porin
999	All	Peptidase

1001	All	Outer membrane protein YaeT
1002	All	Hypothetical protein
1116	UMN179 and 12656/12	Ferrichrome outer membrane transporter
1211	All	Lipoprotein NlpD
1226	All	Outer membrane channel protein GtxE
1268	All	Vitamin B12/cobalamin outer membrane transporter
1346	UMN179 and 12656/12	Putative hemagglutinin
1480	All	LPS-assembly lipoprotein RlpB
1489	All	Outer membrane porin HofQ
1529	All	Phospholipase A
1641	UMN179	Site-specific tyrosine recombinase XerC
1719	All	Surface antigen protein
1875	All	Outer membrane protein
1876	All	Periplasmic chaperone
1928	All	Organic solvent tolerance protein
1939	All	Conserved hypothetical protein
2049	All	Peptidoglycan-associated outer membrane lipoprotein
2156	All	Long-chain fatty acid outer membrane transporter
2165	All	Outer membrane protein
2166	All	Outer membrane phosphoporin
2206	All	Outer membrane protein
2244	UMN179 and 12656/12	Putative hemagglutination
2245	UMN179 and 12656/12	Putative hemolysin secretion/activation protein
2262	UMN179 and 12656/12	Putative hemolysin secretion/activation protein
2263	UMN179 and 12656/12	Outer membrane receptor FepA
2307	All	Enterobactin-like receptor protein
2437	UMN179	Putative adhesin
2445	UMN179	Putative hemagglutinin
